# Supplementary figures and images for: Additive manufacturing of a 3D-segmented plastic scintillator detector for tracking and calorimetry of elementary particles
Source: Commun Eng. 2025 Mar 5;4:41. doi: 10.1038/s44172-025-00371-z (PMC11882974; doi:10.1038/s44172-025-00371-z)

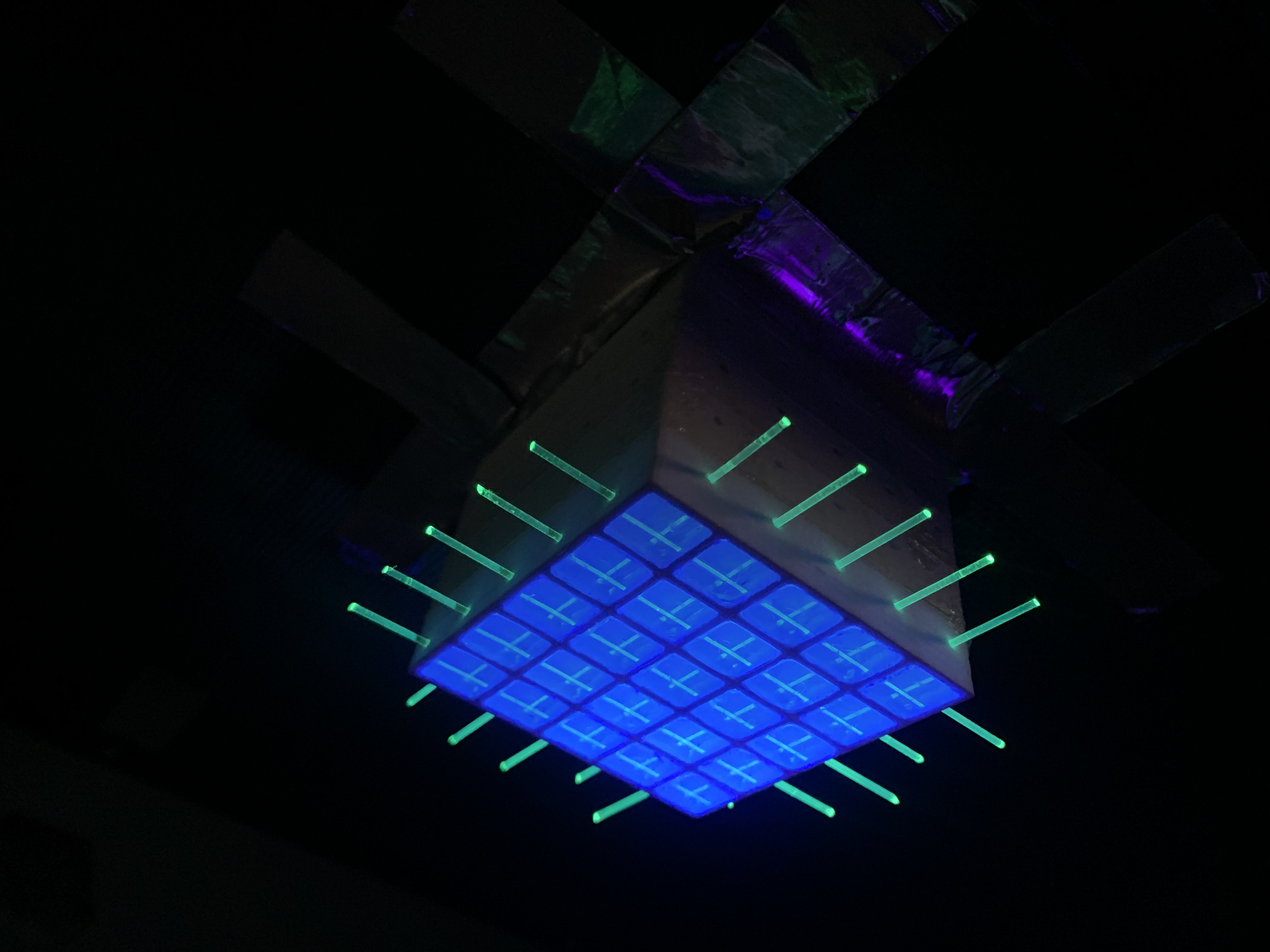

Supplement: Supplementary file 2 — Suggested featured image [file 44172_2025_371_MOESM2_ESM.jpg]
